# Red blood cell transfusion and mortality effect in aneurysmal subarachnoid hemorrhage: a systematic review and meta-analysis protocol

**DOI:** 10.1186/s13643-015-0035-1

**Published:** 2015-04-03

**Authors:** Shane W English, Michaël Chassé, Alexis F Turgeon, Alan Tinmouth, Amélie Boutin, Giuseppe Pagliarello, Dean Fergusson, Lauralyn McIntyre

**Affiliations:** Department of Medicine (Critical Care), The Ottawa Hospital, Civic Campus, 1053 Carling Avenue, Rm F202, Ottawa, ON K1Y 4E9 Canada; Centre for Transfusion Research, Clinical Epidemiology Program, Ottawa Hospital Research Institute, 501 Smyth Road, Box 201B, Ottawa, ON K1H 8L6 Canada; Department of Anesthesia (Critical Care), Hôpital L’Enfant-Jésus, 1401, 18e Rue, H-037, Québec, QB G1J 1Z4 Canada

**Keywords:** Aneurysm, Anemia, Subarachnoid hemorrhage, Transfusion, Red blood cell

## Abstract

**Background:**

Aneurysmal subarachnoid hemorrhage (aSAH) is a devastating disease that leads to important morbidity and mortality in a young patient population. Anemia following aSAH is common and may be exacerbated by the treatments instituted by clinicians as part of standard care. The role and optimal thresholds for red blood cell (RBC) transfusion in this patient population remains unknown.

**Methods/design:**

We will conduct a systematic review of the literature using MEDLINE, EMBASE, and EBM Reviews (including Cochrane Central databases) using a comprehensive search strategy for observational and interventional studies of RBC transfusion in aSAH. Our primary objective is to evaluate the association of RBC transfusion with mortality in aSAH patients. Secondary objectives include a) determining associations between RBC transfusion and poor neurologic outcome, b) defining an optimal RBC transfusion threshold in aSAH patients, and c) describing complications associated with RBC transfusion in aSAH patients. We plan a descriptive reporting of all included citations including study characteristics, methodological quality, and reported outcomes. Clinical and statistical heterogeneity observed between studies will be described. If appropriate, meta-analyses of suitable studies and interpretation of their results will be performed. Effect measures will be converted to obtain relative risks and odds ratios (RR and ORs) with 95% confidence intervals and pooled according to study design (randomized trials and observational studies respectively) using a random effects model.

**Discussion:**

This review will summarize the existing observational and trial evidence regarding RBC transfusion in aSAH patients. The analytical plan has made considerations for different study designs, both observational and interventional in nature, and will summarize the best available evidence to inform the end user and policy and guideline producers and to highlight areas in need of further study.

**Systematic review registration:**

PROSPERO CRD42014014806

## Background

Aneurysmal subarachnoid hemorrhage (aSAH) has an estimated incidence rate of 10 per 100,000 patient years in the general population [1,2] and is a common neurologic cause for intensive care unit (ICU) admission [3]. It affects a relatively young population [4] and thus accounts for significant potential years of meaningful life lost [5] given the important morbidity and mortality the disease imports. Mortality remains high affecting almost 50% of the patients afflicted with aSAH [1,4,6-8].

The period that follows the acute hemorrhage is fraught with multiple possible complications. Anemia is common, affecting more than 50% of aSAH patients [9,10], and is independently associated with poor outcome after SAH [11,12], regardless of SAH severity [13]. Although anemia is defined by the World Health Organization as a hemoglobin of less than 130 g/L in men and 120 g/L in women [14], anemia is typically considered clinically relevant in SAH populations when moderate (or ≤100 g/L) in both [9,10]. Anemia develops a mean of 3.5 days from admission [9], which coincides with the typical onset of another important complication, vasospasm.

Vasospasm is seen in 50% to 75% of patients with aSAH [4,15] leading to ischemic injury or delayed ischemic neurologic deficits (DINDs) from reduced cerebral perfusion. Standard of care management of vasospasm includes hyperdynamic therapy [4], which traditionally included hypertension, hypervolemia and hemodilution (the so-called ‘HHH therapy’). By its very nature, it may lead to or exacerbate anemia. Interestingly, there appears to be stronger data supporting hypertension as being the most beneficial aspect of hyperdynamic therapy in maintaining adequate cerebral perfusion [16] (and hence oxygen delivery) while hemodilution, and its effects on hemoglobin concentration, is considered to be potentially harmful [17].

Recent techniques of micropipetting and monitoring of brain tissue partial pressure of oxygen bring evidence to suggest brain tissue at risk from ischemia benefit from improved oxygen delivery [18,19]. Observational work has shown that brain tissue partial pressure of oxygen is higher with higher hemoglobin concentrations [19] and increases with red cell transfusion [20]. However, transfusion in critically ill patients remains a hotly debated issue. The landmark TRICC trial drastically changed how critically ill patients are managed with respect to RBC transfusion with their finding that a restrictive transfusion strategy (Hb trigger of ≤70 g/L) was as safe as a liberal strategy (Hb trigger ≤100 g/L) and trended towards better outcome [21]. This study, however, is not necessarily generalizable to an aSAH population, as very few patients with neurologic diagnoses were included. Observational work in aSAH has shown that both anemia and red cell transfusion are each risk factors for poor outcome [12,22]. Further, evidence is conflicting as to which of anemia or transfusion carries the bigger risk, or if a risk exists at all [12,23-26]. A recent systematic review examined transfusion thresholds among all neurocritical care patients but was limited to those studies that compared at least two different thresholds [27]. As such, only six studies met inclusion for this review, only one of which included aSAH patients. Our knowledge synthesis will examine the current clinical evidence around anemia and RBC transfusion thresholds and the effect of transfusion on death, neurologic outcomes, and transfusion adverse events among critically ill patients with aSAH.

### Objectives

The primary objective of this review is to evaluate the association of RBC transfusion with mortality in aSAH patients. Mortality will be assessed as reported at hospital discharge, 30 days, and 3, 6, and 12 months. Secondary objectives include 1) determining associations between RBC transfusion/liberal transfusion strategy and poor neurologic outcome (as measured by any of modified Rankin Scale (mRS) ≥4, Glasgow Outcome Scale (GOS) ≤3, or extended Glasgow Outcome Scale (eGOS) ≤4), 2) defining an optimal RBC transfusion threshold in aSAH patients, and 3) describing transfusion adverse events associated with RBC transfusion in aSAH patients.

## Methods/design

This review will be conducted in accordance with The Cochrane Collaboration [28] principles for Systematic Reviews and reported following the PRISMA guidelines [29].

### Search strategy

Our search strategy will be conducted using MEDline + MEDline In-Process & Other Non-indexed Citations, EMBASE Classic + Embase, and EBM Reviews (including Cochrane Central databases) from inception to the moment of review (see Appendix). EMBASE also includes the abstract publications from major international conferences including the International Stroke Conference, Neurocritical Care Society Meeting, Society of Critical Care Medicine, and the International Symposium on Intensive Care and Emergency Medicine. A comprehensive search strategy will be constructed and implemented by a health information specialist with systematic review experience, in collaboration with the research team. MeSH terms will be used to capture each of the principal elements of the research question. To be as inclusive as possible, the strategy will be restricted to focusing on population (aSAH patients) and intervention/exposure (RBC transfusion) and will not be limited by outcome studied. So as to not overlook any possible study for inclusion, our search strategy will include anemia as a search term, separate from transfusion. Our study intervention of RBC transfusion will be targeted by applying inclusion and exclusion criteria as described below. Upon completion, identified citations will be exported to a citation manager (Mendeley Desktop, Mendeley Ltd., v. 1.12.2) for study selection. Manual review of the reference lists of all included studies and previous systematic reviews will be conducted. A final grey literature search will be conducted using ‘Google Scholar’ as well as a review of the trial register (clinicaltrials.gov) for any ongoing and unpublished studies. No language restriction will be utilized in any of the searches. Duplicate citations will be removed. The search strategies will be kept up to date to the time of the end of the review.

### Study screening and inclusion

Both observational (cohort, cross-sectional) and interventional studies will be considered. We will include all retrospective and prospective studies with the goal of detailing all of the available evidence regarding RBC transfusion in aSAH patients. We aim not only to consider comparative studies of different transfusion thresholds as has been done in the past [27] but also to include studies that compare exposed from non-exposed patients.

An iterative process for study selection will be followed using the criteria set out in Table 1. Specifically, we will include all interventional or observational studies that report on an adult (age ≥18 years) hospitalized aSAH population (population), examine RBC transfusion (intervention) and compare either two or more different thresholds or to a non-transfused group (comparator), and report on any clinical outcome (outcome). We will exclude any study which reports exclusively on a pediatric population (age <18 years), non-human studies, and any duplicate or sub-study of previously published (and included) cohorts. All records will first be screened by title and abstract. All citations clearly not relevant to the review (for example, wrong population, pre-clinical study, narrative review) will be excluded. This process will be performed in duplicate by two independent reviewers. Any citation in which an abstract is not available and where suitability for inclusion is questioned will proceed to the next stage. All citations not excluded in the first screen will have full articles retrieved for a second review, in duplicate by independent reviewers, and the selection criteria applied. Any differences in classification between the two independent reviewers will be reviewed and consensus decision made. A third independent senior reviewer will be used in any instance in which consensus is not reached.

**Table 1:** **Study selection criteria**

**Inclusion criteria**	**Exclusion criteria**
1. Study design: interventional or observational	1. Studies of exclusively pediatric population (age <18 years)
2. Included population: hospitalized aSAH patients	2. Non-human studies
3. Included intervention: RBC transfusion	3. Duplicates or ‘sub-cohorts’ of previously published cohorts
4. Comparator: a different transfusion threshold or no transfusion	
5. Study outcome: any	

Our study free-form question is: In adult patients (age ≥18 years) with acute aSAH, is RBC transfusion or a liberal RBC transfusion strategy associated with increased all-cause mortality? We will deem the intervention arm (for interventional studies) or exposure (for observational studies) to be the liberal transfusion strategy or any RBC transfusion respectively administered during the initial hospitalization for aSAH. We will compare this to a restrictive transfusion strategy or non-transfused patients with aSAH. Our primary outcome is all-cause mortality. We will examine hospital mortality and mortality at 30 days as well as 3, 6, and 12 months. We will also examine the following secondary outcomes: 1) poor neurologic recovery (modified Rankin Scale ≥4, GOS ≤3, eGOS ≤4) based on the last follow-up time point, at hospital discharge, and at 3, 6, and 12 months; 2) hospital and ICU length of stay; 3) optimal RBC transfusion threshold; 4) transfusion adverse events; and 5) vasospasm and cerebral infarct incidence.

### Data extraction

A data extraction form will be prepared *a priori* and piloted prior to duplicate extraction by two independent reviewers. Data extraction will include:

- *Study characteristics, design, and methods*: title, authors, journal/source, year and language of publication, country, type of study, study period, total number of patients, case ascertainment and/or inclusion/exclusion criteria, randomization, allocation concealment, and blinding methods (where applicable)
- *Sample characteristics*: age, sex, admission diagnosis, aSAH grade, aneurysm size and location, comorbidities, and baseline hemoglobin
- *Interventions and co-interventions*: aneurysm clip or coil procedures, vasopressor use, mechanical ventilation, externalized ventricular drain (EVD), hyperdynamic (or ‘HHH’) therapy, and RBC transfusion
- *Outcome*: study-specific outcomes as defined by the authors will be captured. In addition, we will abstract nadir hemoglobin, time to nadir hemoglobin, pre-transfusion hemoglobin, ICU admission, clinical complications (including vasospasm and infarction), functional recovery (including mRS, GOS, and eGOS), mortality, and other adverse events

### Analysis plan

A description of all included studies, including demographic, clinical, and methodological quality (see risk of bias), will first be reported with the aid of tables and text. Our cursory review of the literature and a recent narrative review [10] suggests that several observational studies exist that will be the focus of this review. Meta-analyses of observational studies are at particular risk of bias and confounding [30]. Therefore, suitability for meta-analysis will be determined by the degree of heterogeneity (clinical and statistical) observed between the studies. Statistical heterogeneity will be described using the *I*^2^ statistic.

#### Primary outcome

We anticipate that the primary outcome, all-cause mortality, may be reported differently according to the study design. Authors may report the risk of mortality according to exposed/not exposed, using a threshold strategy or a cumulative exposure. Where possible, we will collect the crude numbers of dead and alive patients in each respective group (for example, exposed/non-exposed) at the latest follow-up time point per the study-specific design as well as their associated crude and adjusted effect measures including relative risk (RR), odds ratio (OR), and hazard ratio (HR). Should a meta-analysis be deemed appropriate, effect measures will be converted to obtain RRs for RCTs and ORs for observational studies with 95% confidence intervals (CIs) and pooled according to study design (for example, RCTs vs observational studies, transfused vs non-transfused, comparative threshold studies). Given that we anticipate a certain degree of heterogeneity, a random effects model will be used. Statistical heterogeneity will be reported using the *I*^2^ test with 95% confidence interval.

#### Secondary outcomes

Secondary outcomes will be a combination of dichotomous, ordinal, and continuous measures. Effect estimates of dichotomous outcomes will be presented as RR or ORs and 95% CIs. If appropriate, we will perform meta-analysis, and data presented as a RR will be converted to OR where possible. Neurologic outcomes (mRS, GOS, and eGOS) are expected to be presented either as ordinal data or may have already been dichotomized by the authors. Where possible, ordinal neurologic outcomes will be utilized. All continuous outcome variables will be described with means or medians and associated standard deviations or interquartile ranges as appropriate. Summaries of continuous data will be presented as mean differences with 95% confidence intervals.

##### Optimal transfusion hemoglobin threshold

To describe an optimal hemoglobin transfusion threshold, studies will be grouped according to whether different transfusion thresholds were assessed or if pre-transfusion hemoglobin levels were reported. When a transfusion threshold is the intervention of interest, we will group the results of the lower and higher thresholds for comparison. We will pool the results from studies using similar thresholds (for example, hemoglobin within 10 g/L). If our review includes a sufficient number of studies that assess thresholds in regard to a specific outcome, a meta-regression analysis will be performed to assess the risk of that outcome in regard to the different reported thresholds [31]. In the event of reporting of ranges of pre-transfusion hemoglobin, the exposure will be assigned as the midpoint of the range.

#### Risk of bias

Risk of bias will be assessed using the Downs and Black tool [32] for observational studies and the Cochrane Collaboration tool for assessing the risk of bias in RCTs [33]. Bias risk assessment will be completed in a similar fashion as the study selection process: in duplicate by two independent assessors. Cases of discordance not resolved by consensus will be reviewed by a third senior assessor. Risk of bias assessment of all included studies will be summarized and presented in table format. Meta-analysis, if possible, will be performed including all studies, with a planned sensitivity analysis (see below) to be performed using only those studies at low risk of bias. Low risk of bias will be defined as those studies with a score of ≥25 using the Downs and Black tool or those deemed low risk across all domains of the Cochrane Collaboration’s tool for assessing risk of bias. The authors recognize that no formal cutoffs exist to define low or high risk of bias with the Downs and Black tool (written correspondence with the author); however, we deem that in order for low risk of bias to exist in an observational study, there must be excellent reporting, high internal and external validity with little risk of confounding, and sufficient study power (all domains assessed with this tool) such that high scores in each of these domains are necessary to meet low-risk criteria.

##### Subgroup analyses

Pre-planned subgroup analyses to examine clinical heterogeneity will include transfusion in anemic patients (hemoglobin ≤100 g/L), first transfusion pre/post vasospasm and transfusion in high-grade aSAH patients (defined as Hunt and Hess grades 4 to 5, and/or Fisher grade 4, WFNS grades 4 to 5), and open surgical clipping versus endovascular coiling.

##### Sensitivity analyses

To test the robustness of our findings, we plan the following sensitivity analyses: 1) studies with low versus unclear/high risk of bias, 2) studies with study periods after 2005 (the year of the ISAT trial [34] publication which resulted in a management shift from surgical clipping to interventional coiling of certain aneurysms) versus before 2005, and 3) studies in which important confounders of RBC transfusion and mortality, such as aSAH severity, pre-transfusion and hemoglobin nadir, and vasospasm, are controlled for in their primary analysis.

## Discussion

Subarachnoid hemorrhage is a devastating event often with lasting effects that greatly impact functionality and quality of life. The natural history of subarachnoid hemorrhage often includes complications like vasospasm that leads to further injury from ischemic damage whose very treatment may lead to hemodilution, potentially further compromising oxygen delivery. This rather uniquely sets apart this population from others in which a restrictive transfusion strategy has been shown to be at least as safe as a liberal one, and perhaps superior [21,35,36]. A restrictive transfusion strategy is not clearly beneficial for SAH patients, and doubt has been cast with small interventional studies involving other end-organ ‘at-risk’ populations [37].

This review proposes to systematically identify, gather, and summarize the observational and trial evidence that exists regarding RBC transfusion in SAH patients, using a rigorous methodology. We aim to assess the effect of RBC transfusion on all-cause mortality in addition to other clinically important outcomes which will serve as a summation of the evidence to best inform clinical decisions at the bedside.

We anticipate that the majority of available evidence will be the results of observational work rather than randomized controlled trials. As such, we have planned for a large descriptive component of this review which will include tables, figures, and charts. We recognize that the risk of bias is much higher, particularly in retrospective observational studies and as such have incorporated into the protocol two well-recognized and accepted assessment tools in addition to planned assessments of clinical and statistical heterogeneity. The results from observational studies may be inflated as was the experience in a recent systematic review of large-scale observational studies assessing the effect of transfusion on mortality in a heterogeneous patient population [38]. Although their findings were consistent across studies despite varying study designs and degrees of confounding adjustment, the magnitude of the findings were significantly larger with the observational studies. Nonetheless, clinicians are often forced to turn to observational studies to inform practice, since well-conducted randomized controlled trials are very limited in number. In fact, in SAH, the vast majority of the related recommendations in the most recent guidelines are based on observational evidence or expert opinion alone [39]. This review will formulate the best available evidence, which is dependent on the quality of the existing evidence. Careful interpretation of the findings, in light of identified limitations, is essential.

This knowledge synthesis thus will not only serve to inform the end user (that is, the clinician) but also policy and guideline producers. Finally, this review is essential to further highlight areas in need of further study. It will inform the creation of other scientific questions and help formulate other research protocols.

## Appendix

### Search strategy

The following databases will be used to conduct our search strategy:

1. MEDline + MEDline In-Process & Other Non-indexed Citations (1946 to present)
2. EMBASE Classic + Embase (1947 to present)
3. EBM Reviews (incl Cochrane) (2005 to present)
4. Search of trial registers for ongoing and unpublished studies

### SAMPLE *search strategy*

Database: Embase Classic + Embase <1947 to present>, Ovid MEDLINE(R) In-Process & Other Non-Indexed Citations and Ovid MEDLINE(R) <1946 to present>

Search strategy:

1. exp Subarachnoid Hemorrhage/ (44404)
2. *intracranial hemorrhages/ or *cerebral hemorrhage/ or *vasospasm, intracranial/ (45036)
3. *Intracranial Aneurysm/ (27317)
4. *Rupture, Spontaneous/ or *rupture/ or rupture$.tw. (207853)
5. 3 and 4 (7647)
6. *Aneurysm, Ruptured/ (8661)
7. exp *brain/ or exp *meninges/ (1256700)
8. 6 and 7 (430)
9. ((subarachnoid or arachnoid$) adj3 (haemorrhag$ or hemorrhag$ or haematoma$ or hematoma$ or bleed$ or blood$)).tw. (40217)
10. ((brain or cereb$ or intracranial) adj3 aneurysm$ adj3 ruptur$).tw. (6251)
11. ((brain or cereb$ or intracranial) adj3 aneurism$ adj3 ruptur$).tw. (30)
12. ((cerebral or intracranial or cerebrovascular) adj6 (vasospasm or spasm)).tw. (8794)
13. sah.tw. (15075)
14. 1 or 2 or 5 or 8 or 9 or 10 or 11 or 12 or 13 (101951)
15. Erythrocyte Transfusion/ (18728)
16. ((red blood cell$ or rbc or erythrocyte$ or red cell$) adj2 (transfus$ or therap$)).tw. (14636)
17. *Blood Transfusion/ (67415)
18. rbct.tw. (125)
19. (blood adj2 transfus$).tw. (89864)
20. (hemotransfus$ or haemotransfus$).tw. (522)
21. or/15-20 (148295)
22. 14 and 21 (371)
23. *anemia/ or anemia.tw. or anaemia.tw. (267609)
24. 14 and 23 (498)
25. *Hemoglobins/ or hemoglobin$.ti. orhaemoglobin$.ti. (100884)
26. 14 and 25 (265)
27. 22 or 24 or 26 (1012)
28. animals/ not humans/ (5123176)
29. 27 not 28 (949)
30. **29 use emczd (622) EMBASE**
31. exp Subarachnoid Hemorrhage/ (44404)
32. intracranial hemorrhages/ or cerebral hemorrhage/ or vasospasm, intracranial/ (94172)
33. Intracranial Aneurysm/ (33870)
34. Rupture, Spontaneous/ or rupture/ or rupture$.tw. (227167)
35. 33 and 34 (9450)
36. Aneurysm, Ruptured/ (15457)
37. exp brain/ or exp meninges/ (2087107)
38. 36 and 37 (1927)
39. ((subarachnoid or arachnoid$) adj3 (haemorrhag$ or hemorrhag$ or haematoma$ or hematoma$ or bleed$ or blood$)).tw. (40217)
40. ((brain or cereb$ or intracranial) adj3 aneurysm$ adj3 ruptur$).tw. (6251)
41. ((brain or cereb$ or intracranial) adj3 aneurism$ adj3 ruptur$).tw. (30)
42. ((cerebral or intracranial or cerebrovascular) adj6 (vasospasm or spasm)).tw. (8794)
43. sah.tw. (15075)
44. 31 or 32 or 35 or 38 or 39 or 40 or 41 or 42 or 43 (145988)
45. Erythrocyte Transfusion/ (18728)
46. ((red blood cell$ or rbc or erythrocyte$ or red cell$) adj2 (transfus$ or therap$)).tw. (14636)
47. Blood Transfusion/ (145644)
48. rbct.tw. (125)
49. (blood adj2 transfus$).tw. (89864)
50. (hemotransfus$ or haemotransfus$).tw. (522)
51. or/45-50 (199301)
52. 44 and 51 (1576)
53. anemia/ or anemia.tw. or anaemia.tw. (321603)
54. 44 and 53 (1752)
55. Hemoglobins/ or hemoglobin$.ti. orhaemoglobin$.ti. (207188)
56. 44 and 55 (1173)
57. 52 or 54 or 56 (3847)
58. animals/ not humans/ (5123176)
59. 57 not 58 (3698)
60. **59 use prmz (625) MEDLINE**
61. 30 or 60 (1247)
62. remove duplicates from 61 (957)

## Abbreviations

aSAH: aneurysmal subarachnoid hemorrhage
CI: confidence interval
DIND: delayed ischemic neurologic deficit
eGOS: Extended Glasgow Outcome Scale
EVD: externalized ventricular drain
GOS: Glasgow Outcome Scale
HHH therapy: hypertension, hypervolemia, hemodilution therapy
HR: hazard ratio
ICU: intensive care unit
mRS: modified Rankin Scale
OR: odds ratio
RBC: red blood cell
RCT: randomized controlled trial
RR: relative risk
SAH: subarachnoid hemorrhage
WFNS: World Federation of Neurosurgeons
